# Supplementary material for: Patterns of Intron Gain and Loss in Fungi
Source: PLoS Biol. 2004 Nov 30;2(12):e422. doi: 10.1371/journal.pbio.0020422 (PMC532390; doi:10.1371/journal.pbio.0020422)
Supplement: Table S1 — Also available at http://genes.mit.edu/NielsenEtAl/. (4.3 MB ZIP). [file pbio.0020422.st001.zip › NielsenEtAl/html/483.html]

AN3515.1.NCU06522.1.MG07546.1.FG10661.1


```
 CLUSTAL W (1.82) Multiple Sequence Alignments - Introns Inserted


Sequence 1: NCU06522.1	560 aa
Sequence 2: MG07546.1	549 aa
Sequence 3: FG10661.1	558 aa
Sequence 4: AN3515.1	530 aa
Alignment Length: 580 aa
Number Identitical Residues: 271 aa
Alignment Score (without introns) 14061


MG07546.1 	MASNREATAG-ADAEKSPTMAPPRNVDMDTSPSSLALEAKHAASSEQEMTLWAAIKTYPK
NCU06522.1	MADQKPLTSSPEEATEAGTTTTTTIPQAQIQDDVVLVEAKHAADTEHEMTLLRAIQLYPK
FG10661.1 	MASPKPFPEKQPNAQHDEAVTP----------ETLASEAKVAAEAEHYMTLWEAVKTYPK
AN3515.1  	MENHDPQPKQ------------------------ELAHGQQGARQEQGMSLWQGLRLYPK
          	* .    .                             ..: .*  *: *:*  .:: ***

MG07546.1 	AIGWSVLLSSTLIMEGYDLALLSSLYNSPVFNQKYGEFNPATGKWVISAAWQSGLSNGAR
NCU06522.1	AIGWSVLLSSTLIMEGYDLALLGSLYASPVFNKKFGELNPATGKYAVSAAWQSGLSNGAR
FG10661.1 	AIGWSVLLSTTLIMEGYDLALLGNLYASPVFNEKFGTYDAKKEKFAVSAAWQSGLSNGAR
AN3515.1  	AVAWSVLLSSTLIMEGYDLALLGSLYASPAFNQRYG-VQAANGDWGVPASWQSALSNGAR
          	*:.******:************..** **.**:::*  :. . .: :.*:***.******

MG07546.1 	AGEILGLILAGWASDRWGYRVTTMLGLVLMMAFIFVLFLAPSVEVLVLGEVLCG~-----
NCU06522.1	AGEIFGLIFAGWAADRIGYKKTTIASLVLMITFIFVLFFAPNVKVLVVGEVLCG1-----
FG10661.1 	AGEIIGLILAGWASDRYGYKMTTVGALVLMIAFVFVLFFAPNIKILVLGEVLCG~TYLSV
AN3515.1  	VGEVIGLIISGFVSERLGYRWTMIYALIAMNAVIFLFFFAVNVQMLLSAEILAG~-----
          	.**::***::*:.::* **: * : .*: * :.:*::*:* .:::*: .*:*.*      

MG07546.1 	-----------IPWGAFQSVTPAYASEVAPVALRPYLTTFINMCWVVGQFLAQAVNYNSI
NCU06522.1	-----------IPWGAFQSVTPAYASEVAPMVLRPYLTTFINMCWVIGQFFAAAVNKGSV
FG10661.1 	DYKYTFADETGIPWGAFQSVTPAYASEVAPVVLRPYLTTFINMCWVIGQFFAAAVNKGSV
AN3515.1  	-----------IPWGIFQTLPAAYASEVCPVVLRPYLTTYINMCWVMGQFIAAGVNRGSF
          	           **** **::..******.*:.*******:******:***:* .** .*.

MG07546.1 	RRTDEWAYRIPFGVQWVWPVPILVGVWFAPE~SPWWFVRHGDRAAAKRSLLRLTSPGQPG
NCU06522.1	GRTDQWAYRIPFGVQWVWPIPILAGLVFAPE~SPWWYVRHDRREEAKQSLLRLTSRNQPG
FG10661.1 	GRGDEWAYRIPFGVQWVWPVPILAGVIFAPE2SPWWHVRKGNRSAAKKSLLRLTSPQQPN
AN3515.1  	QRMDQWSYRVPFAVQWAWPLPILTGILFAPE~SPWWHVRRGDIARAKHALQRLTSPQDAS
          	 * *:*:**:**.***.**:***.*: **** ****.**:.    **::* ****  :..

MG07546.1 	FDADETIAMIEHTNELERRVKEGVRFRDCFRGVDLRRTEVVVGIWLVQTLGGQNLMGYFS
NCU06522.1	FNVDETLAMIEHTNELEKQMKEGVTFRDCFRGVDLRRTEIVVGIWLVQTLGGQNLMGYFS
FG10661.1 	FNADETIAMIEHTNEMEKNLKEGTSYRDCFKGVDLRRTEIVIGIWLVQTLGGQNLMGYFS
AN3515.1  	FDADATIAMIQHTNELEKSLSAGTSYLDCFRGVNRRRTEIVCGIWIVQTLSGQNIMGYFA
          	*:.* *:***:****:*: :. *. : ***:**: ****:* ***:****.***:****:

MG07546.1 	YFLTQAGMDASNSLALSMGQYALGMVGTAGSWLLMARVGRRTIHFAGLTAQLLILLVVGG
NCU06522.1	YFLTQAGMDPSNSFSLSMGQYALGMVGTAGSWFLMSRVGRRRIHFSGLCAQFCLLAIVGC
FG10661.1 	YFLTQAGMDASNSFSLSMAQYALGMVGTFGSWFLMARVGRRTIHFTGLCTQLVILIIVGS
AN3515.1  	YFCVQAGLPEIHSFNLSLGQYALGVLGTIGSWFLMSALGRRTIHLLGLSLLFTLLIITGS
          	** .***:   :*: **:.*****::** ***:**: :*** **: **   : :* :.* 

MG07546.1 	LAFA--GTNAGVWAIGGMLIAFTFVYDFTVGPVTYSLVSELSSTRLKAKTIVSARAAYNA
NCU06522.1	LSFG--GNKASVWAIGGVLVAFTFVYDFAVGPVTYCLISELSSTRLKAKTIVLARAAYNA
FG10661.1 	LSFA--NNNGSVWAIGAMLIVFTFVYDFTVGPVTYSLISELSSTRLKAKTIVMARAAYNA
AN3515.1  	LSFAPDSNNAAKWAIGAMLMVFTLVYDISIGPVTYSLVSELSSTRLKAKTINLARAGYNI
          	*:*.....:.. ****.:*:.**:***:::*****.*:*************  ***.** 

MG07546.1 	SNIFVNVMTNYQLSSASTGWNWGAKTAFFWAGTAALSAVWVFFRVPEPKGRTYAELDILF
NCU06522.1	SNIFVNVMTNYQLS--STAWNWGARTAFFWAGSCLLSAIWVFFRLPEPKGRTYAELDILF
FG10661.1 	SNIFVNVMTNYQLS--SSAWNWGARTAYFWAGTCLLSAIWVYFRLPEPRNRTYAELDVLF
AN3515.1  	SNIVVNVLTNYQLN--DSAWAWGARSAYFWAGTCLLCLCWSAMRVPEPKGRTYEELDLLF
          	***.***:*****.  .:.* ***::*:****:. *.  *  :*:***:.*** ***:**

MG07546.1 	EQGVSARKFASTKIDPY---EHGVESTAKEEQV---------
NCU06522.1	EKKVTARKFRKTKVDPFDLRSHGEKKMSLEKMESHEGKEIEA
FG10661.1 	EKRVSARKFAQTEVDPYSHSVISDKKPTSEKEIQ--------
AN3515.1  	EQRVPAWRFKKTAVDPYQGLAETEGHVREDQQKSS-------
          	*: *.* :* .* :**:.           ::  .
```
